# Lipid Nano-System Based Topical Drug Delivery for Management of Rheumatoid Arthritis: An Overview

**DOI:** 10.34172/apb.2023.075

**Published:** 2023-04-29

**Authors:** Komal Diliprao Dhule, Tanaji Dilip Nandgude

**Affiliations:** Dr. D. Y. Patil Institute of Pharmaceutical Science and Research, Pimpri, Pune 411018, Department of Pharmaceutics, Pune, Maharashtra, India.

**Keywords:** In vivo animal models, Lipid nanocarriers, Patents, Rheumatoid arthritis

## Abstract

The overall purpose of rheumatoid arthritis (RA) treatment is to give symptomatic alleviation; there is no recognized cure for RA. Frequent use of potent drugs like non-steroidal anti-inflammatory drugs (NSAIDs) and disease-modifying antirheumatic drugs (DMARDs), lead to various adverse effects and patient compliance suffers. On the other hand, there are many drawbacks associated with traditional methods, such as high first pass, high clearance rate, and low bioavailability. Drug administration through the skin can be a promising alternative to cope with these drawbacks, increasing patient compliance and providing site-specific action. The stratum corneum, the uppermost non-viable epidermal layer, is one of the primary limiting barriers to skin penetration. Various nanocarrier technologies come into play as drug vehicles to help overcome these barriers. The nanocarrier systems are biocompatible, stable, and have a lower cytotoxic impact. The review discusses several lipid-based nanocarrier systems for anti-rheumatic medicines for topical administration it also discusses in-vivo animal models for RA and provides information on patents granted.

## Introduction

 Rheumatoid arthritis (RA) is an autoimmune, inflammatory disorder, characterized by polyarticular swelling in synovium tissue which leads to the destruction of articular cartilage.^1^ RA primarily targets the small diarthrodial joints of the hand and feet.^2^ Immunological cells like macrophages, T, and B cells are found in synovium and involved in the production of several cytokinins, interleukins (IL-1B and IL-6), and tumor necrosis factor (TNF-alpha) on activation.^1^ These inflammatory mediators and cytokine production results in progressive inflammation and joint swelling. Further, the production of TNF-alpha and IL-1B triggers enzyme metalloprotease and osteoclast production which results in bone erosion, and finally destruction of cartilage. This causes severe pain, swelling, bone stiffness, and destruction of functions.^1,3^ The conventional approaches for the management of RA include treatment with disease-modifying antirheumatic drugs (DMARDs) which improve functioning and slow down the process of joint damage. It includes drugs like methotrexate, leflunomide, hydroxychloroquine, and sulfasalazine.^4^ Another treatment approach involves the use of corticosteroids, Non-steroidal anti-inflammatory drugs (NSAIDs; cyclooxygenase 2 inhibitors), and biological response modifiers (infliximab, adalimumab, etanercept, rituximab, abatacept, rituximab, tocilizumab, tofacitinib) for symptomatic action like pain and inflammation reduction.^5^ Although there are many advancements in RA therapies still safety and efficacy concerns limit their use.^1^ Many adverse effects are associated with the drug used as cyclooxygenase-2 inhibitors have been withdrawn due to the serious cardiovascular toxicity associated with their use.^6^ Incidence of gastrointestinal (GI) distress (nausea, abdominal pain, diarrhea), rash/allergic reaction, bone marrow suppression, and hepatotoxicity, are higher with the use of methotrexate, leflunomide, and sulfasalazine. They are also associated with a high risk of cardiovascular disorders, fungal and viral infections with the occurrence of tuberculosis reactivation, herpes zoster, and hepatitis B/C. IL-17 inhibitors have been reported to cause inflammatory bowel disease.^7^ This all complications and adverse effects of drugs turn towards the development of novel carrier systems, with higher efficiency and safety.

 Currently, topical drug delivery has gained wide attention as it is a non-invasive technique, has minimum side effects associated with the GI tract, avoids hepatic first-pass metabolism, and protects drugs from GI instability and enzymatic degradation. The major limitation of topical delivery is the permeation of the drug through the stratum corneum, which forms the uppermost layer of skin.^8^ To overcome these limitation various methods like iontophoresis, sonophoresis, electroporation, and microneedles,^9^ has been used to some extent, coming to the lipid-based nanosystems like nanoemulsion, liposomes, ethosomes, transferosomes, niosomes, and solid lipid nanocarriers are developed which can be proven as a powerful approach.

## Pathogenesis of rheumatoid arthritis

**Figure 1:**
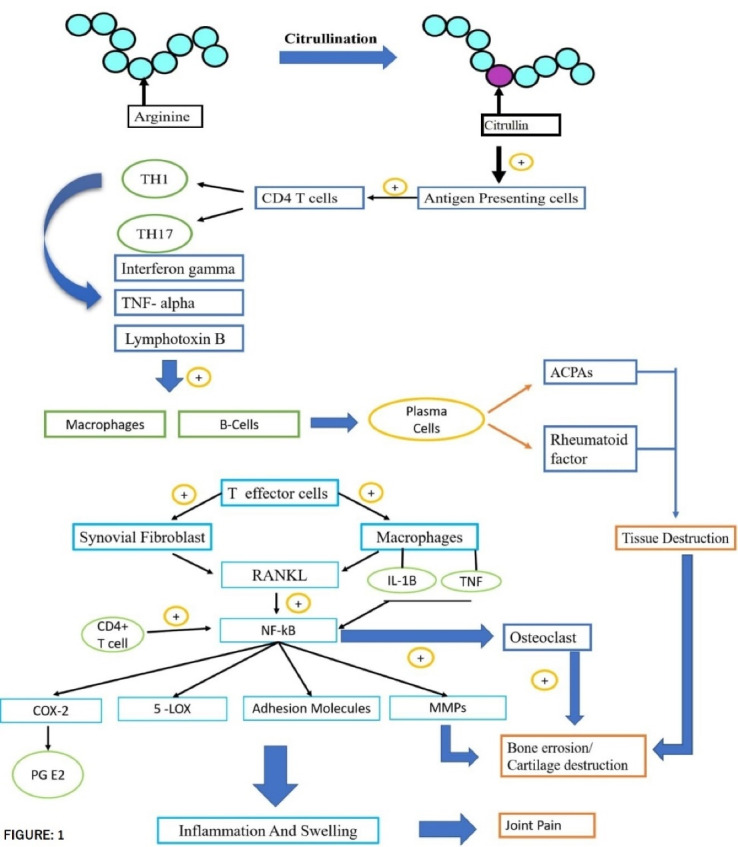
Pathogenesis of rheumatoid arthritis

**Figure 2:**
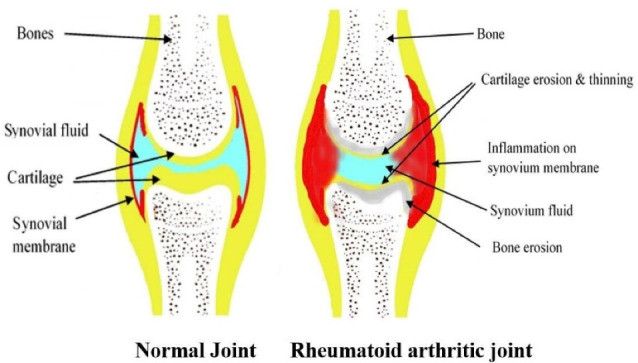
Condition in normal joint and rheumatoid arthritis joint

 Genetic, environmental, and immunologic factors act as a trigger for RA disturbing the immune system functioning. As shown in Figure 1, a post-translational modification of amino acid arginine into citrulline that is citrullination process takes place, which is recognized as foreign antigen by the immune cells and stimulates the development of anticitrullinated peptide antibodies, or anti-cyclic citrullinated peptide (anti-CCP) auto-antibodies.^10^ The citrulline containing protein such as filaggrin, type II collagen, and vimentin is recognized as a foreign antigen. These antigens are picked up by the antigen-presenting cells and are carried to the lymph nodes, where due to interaction between T-cell receptor and class II MHC-Peptide (major histocompatibility complex) antigen CD4 + T cells are activated, which then differentiates into T helper 1 (TH1) and T helper 17 cells (TH17). T H1 secrets Interferon-gamma TNF-alpha and Lymphotoxin B which activates macrophages and B-cells. B-cells then differentiate into plasma cells. These plasma cells secrete auto-antibodies like Rheumatoid factor (anti-IgM antibodies) and anticitrullinated peptide antibodies or anti-CCP antibodies. These T-cells and auto-antibodies enter into circulation reaching to joints. Rheumatoid factor forms a complex with Fc portion of altered IgG antibodies forming immune complexes. Anti-CCP targets citrullinated proteins. Now, this activated system destroys local tissue. Simultaneously T–effector cells stimulate synovial macrophages and fibroblasts to secrete proinflammatory mediators (like TNF-alpha, IL-1, and IL-6) which results in synovial inflammation and promotes angiogenesis to bring more inflammatory cells to the joints. Inflammatory cytokines increase the expression of receptor activator of nuclear factor κB (RANK) ligand (RANKL), on the surface of T-cells which brings about activation of osteoclasts. These activated osteoclasts cause bone destruction.^4^ The matrix metalloproteases secreted by synovial cells are involved in the destruction of cartilage.^8^ The overall pathogenesis, results in bone erosion, cartilage destruction, and inflamed condition. As depicted in Figure 2, the difference between normal and RA joints. The joint with RA shows inflammation on synovium or synovium membrane, thinning of cartilage, continuous erosion of bone and reduction in synovial fluid.

## Conventional treatment for rheumatoid arthritis and their drawbacks

 The widely used drugs of NSAIDs class to relieve pain and inflammation are most likely associated with undesirable side effects like ulcers, internal bleeding, kidney failure, and increased risk of a heart attack. They are also involved in the induction of reactive oxygen species resulting in increased oxidative stress in different tissues.^11^ NSAIDs and COX-2 inhibitors both are equally effective and are associated with an increased risk of GI, renal, and cardiovascular adverse effects.^12^ The study investigated by Combeet al^13^ conclude that long-term use of etoricoxib, a COX inhibitor is associated with a greater risk of thrombotic events, renovascular adverse events, and GI intolerance.^13^ DMARDs plays important role in the treatment of RA, used as monotherapy or given in combination with glucocorticosteroids or NSAIDs drugs.^14^ The first choice from this category is methotrexate, many of these drug molecules like cyclosporine, gold, and D-penicillamine are associated with potential renal toxicity while drugs like methotrexate, azathioprine, antimalarials, sulfasalazine, leflunomide, etanercept, and infliximab have relatively low effect on renal function.^15,16^ They are also associated with side effects like liver and digestive organ dysfunction, stomatitis, and myelosuppression.^16^ Sulfasalazine is mainly associated with adverse GI effects (e.g., nausea, vomiting, dyspepsia, anorexia), CNS effects (e.g., headache and dizziness), and rashes.^17^ The use of biological agents in RA is another treatment approach etanercept, infliximab, and adalimumab targets TNF and IL-1 activity, administered as monotherapy or given in combination with methotrexate.^18^ In addition to their side effects like bacterial and fungal infection, they are also associated with malignancy especially mon-Hodgkin’s lymphoma.^16^

## Herbal medicine approach

 According to the reported study, around 450 plant species belonging to 100 families are being traditionally used for arthritis management.^19^ There are many phytoconstituents or herbs reported for the management of RA some of them are *Boswellia serrata *(frankincense),* Curcuma longa *(turmeric),*Eremostachys laciniata, Eucommia ulmoides, Matricaria chamomilla, Paeonia lactiflora, Withania somnifera*(ashwagandha),* Zingiber officinale,*^20^ *Piper nigrum, Commiphora mukul, Pongamia pinnata, Betula platyphylla,*^19^ *Nigella sativa *(black cumin),*Tinospora cordifolia *(guduchi),* Capsicum frutescens *(cayenne),* Allium sativum *(garlic),* Tripterygium wilfordii*(thunder duke vine).^21^ Other traditional Chinese herbal medicines used in RA treatment are *Angelica Sinensis* Radix, *Paeoniae* Radix Alba, *Ramulus Cinnamomi*, *Glycyrrhizae* Radix et Rhizoma, and *Clematidis* Radix et Rhizoma, which shows improved treatment efficiency when given with DMARDs.^22^ There is very little information available on pharmacodynamics and pharmacokinetic potential of herbal drugs and the incidence of adverse effects like herb-drug and herb-herb interactions.^23^ On oral administration, these herbs may show drug-herb or herb-herb interactions which results in various side effects and may create a potential risk to the patient.^24^ Many of the studies have reported side effects associated with herbal drugs such as GI upset, dizziness, fatigue, male reproductive toxicity, and alteration in the female menstrual cycle. Other factors that limit their use is poor absorption, low bioavailability, rapid metabolism, and elimination of active component.^25^ On the other hand, the presence of multiple components in the herbal drugs, lack of standardization technique, and difficulties in the authentication of species as well as the presence of contaminants make it challenging to use.^23^

 To avoid GI disturbances, first-pass effect, and to provide site-specific action various topical formulations were developed and studied.^26^ One of the studies conducted by Aiyalu et al^27^ involves the development of containing *Cardiospermum halicacabum* and *Vitex negundo* leaf extracts loaded gel followed by evaluation of anti-arthritic activity using Freund’s complete adjuvant induced arthritis method and concluded reduction in paw volume, TNF-α level with no agglutination in C-reactive protein and rheumatic factor, further histopathological examination justified anti-arthritic activity of herbal gel.

###  Lipid-based Nanomedicines as a topical treatment approach for RA

 Recent approaches came up with the use of a topical route that provides non-invasive drug delivery and overcomes the drawbacks and adverse events associated with the conventional route, it also bypasses hepatic first-pass metabolism. Additionally, it is possible to deliver various drug molecules both hydrophilic and lipophilic.^28^ The stratum corneum (non-viable epidermis layer) is 5–20 μm thick, consisting of 10-15 layers of thick corneocytes, lipid matrix, corneodesmosomes, and a tight junction that makes stratum corneum acting as a barrier for drug penetration.

**Figure 3:**
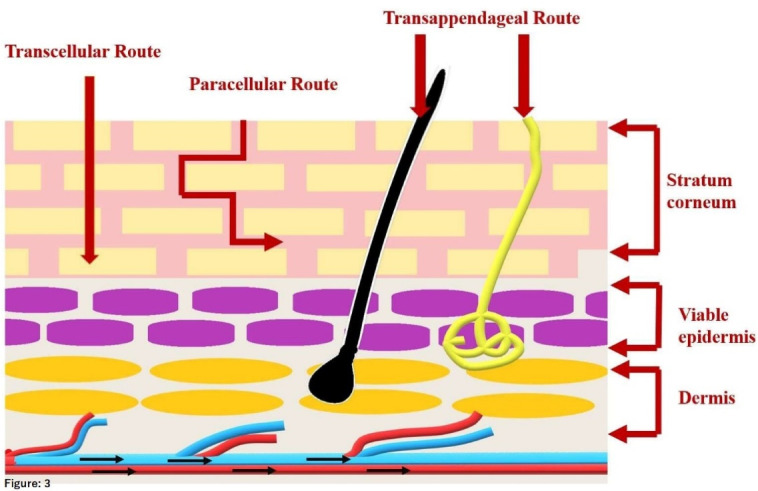
Proposed penetration pathways for lipid carriers to cross skin layers

 On the other hand, as shown in Figure 3 there are some proposed pathways for the molecule to cross the stratum corneum, which includes,

1. Paracellular (intercellular) pathway, the passage of the small lipophilic molecule through the lipid matrix which is in between the corneocytes.
2. Transcellular (intracellular) pathway, the passage of the lipophilic molecule through the corneocytes by partitioning into lipophilic and hydrophilic domains.
3. Transfollicular (shunt) pathway, involves the passage of drug molecules through sweat glands and hair follicles.

**Figure 4:**
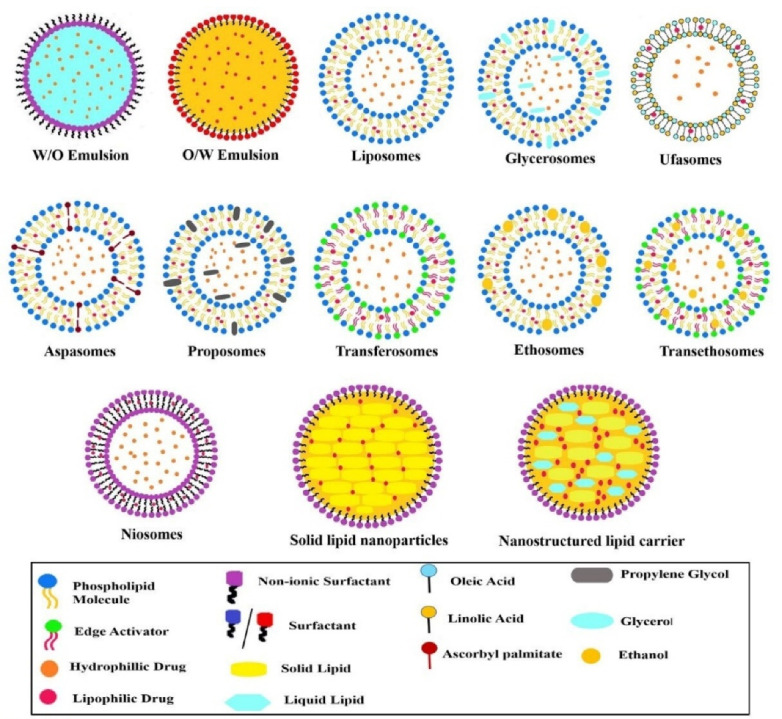
Structural representation of various lipid-based nanocarrier systems

After crossing this non-viable layer (that is stratum corneum) it reaches to viable layer and passing through different layers it distributes into the dermal layer reaching the blood capillaries.^29^ Here, to make the molecule cross the barrier by incorporating it into a suitable carrier. Various nano lipid formulations mentioned in Figure 4, came into the picture which shows better penetration and prolonged residence time as compared to the conventional topical formulations.^8^ In addition these lipids also exhibit biocompatibility, increase adhesiveness with skin which in turn reduces water loss and promote skin hydration, and can also lead to lipid exchange between carriers and the outermost layer of skin, which encourages drug penetration.^30^ These systems are discussed further.

###  Nanoemulsion

 These are thermodynamically stable, biphasic oil-in-water or water-in-oil type of dispersions stabilized by an emulsifier. The particle size of the dispersed phase ranges from 20 to 200 nm. Because of such small particle size, this system appears transparent.^31^ Nanoemulsion with drug delivery plays multiple roles that are the protection of the drug from degradation, carries both hydrophilic and lipophilic molecules in a single system, and can also provide control release action.^32^ Nano-sized dispersed phase in nanoemulsion makes the emulsion stable against sedimentation and creaming.^33^ It is found to be a promising alternative to improve penetration of poorly soluble drugs through the skin, both hydrophilic and hydrophobic drug molecules can be incorporated into it. It is challenging to use nanoemulsion topically due to its low viscous nature and inappropriate spreadability. Incorporating nanoemulsion into a gel can be a potential approach to overcome these challenges. Nanoemulgel is a nanoemulsion-based hydrogel that was developed, which shows improved spreadability and can also act as a drug reservoir to control the release of drug.^34^ Gokhale et al^35^ formulated and evaluated Quercetin-loaded nanoemulsion based gel for managing RA, the study conducted by using HIG-82 and RAW 264.7 cells showed a strong inhibitory effect on LPS-induced TNF-α production with no toxic effect on synoviocytes and also inhibited complete Freund’s adjuvant induced paw edema in rats. Nanoemulsions can be also used for combination therapy, to deliver two drugs simultaneously. One of the studies, conducted by Poonia et al^36^ revealed controlled drug release for up to 48 hr. and potential anti-arthritic activities with 78.76% inhibition in inflammation, accessed in rats when nanoemulsion loaded gel of two drugs that are Methotrexate and Resveratrol in combination, was investigated for transdermal use.

 An advanced form of nanoemulsion drug delivery is the development of a Self-Nanoemulsifying drug delivery system, which has to cope with the limitations and certain stability issues like “Ostwald ripening” associated with the nanoemulsion system.^37^ These systems are a mixture of oil, drug, surfactant, and cosurfactant. These anhydrous preconcentrates of nanoemulsion turns into a nanoemulsion when they come in contact with the aqueous phase.^38^ A study carried out by Badran et al^39^ reveals the development of a self-nano emulsifying drug delivery system loaded with Meloxicam for transdermal application for managing arthritic pain, this formulation has shown droplet size of about 14.41 ± 2.50 nm to 25.58 ± 2.33 nm (ultra-fine) with a higher release rate and 11.89 folds increase in drug permeation compared to control. Nanoemulsion can be a promising carrier system for the delivery of herbal and animal-origin drugs through the skin. A recent study conducted by Abbasifard et al^32^ was based on the development of bee venom nanoemulsion which was evaluated for its penetration and activity to modulate serum levels of endothelin-1 (which is a pro-inflammatory factor that participates in RA pathogenesis) in collagen-induced RA model and found to have good skin permeation and showed a decrease in serum level of endothelin-1, because of anti-oxidant, anti-inflammatory and immunomodulatory effect of bee venom. Loading herbal drug extract in nanoemulsion is also found to be beneficial, Chandra et al,^40^ to reduce the dose of ginger extract, a potent anti-inflammatory agent and to promote its drug delivery through the skin, incorporated ginger extract into a nanoemulsion and formulated as nanoemulgel which has improved transdermal absorption of the ginger extract.^40^

###  Liposomes

 Bilayered phospholipid vesicles, spherical in shape, with a self-closed structure having an aqueous core are called liposomes.^41^ They can encapsulate both hydrophilic and lipophilic molecules. Composed of phospholipids, cholesterol, and stabilizer and classified as unilamellar to multilamellar vesicles depending on their internal structure.^42^ They can serve as ideal carriers for most of the anti-rheumatic drugs because of their ability to show site-specific action, limiting the systemic exposure with low side effects.^43^ Bilayer structure can show fusion with the cell membrane, releasing liposomal contents.^44^ The phospholipids used can also act to increase the penetration through the skin, as it has a similar lipidic composition to that of the skin. They can also serve as a reservoir in the stratum corneum layer producing sustained-release action. Puglia et al^45^ investigated *in vivo* drug release profile of indomethacin-loaded liposomes composed of dipalmitoyl-L-phosphatidylcholine and cholesterol, and found a more prolonged anti-inflammatory effect was observed with liposomal gel as compared to the gel without liposomes. This sustained action was probably because of the interaction of lipid vesicles and lipid in the stratum corneum, producing a depot.One of the studies, investigated toenhance the transdermal RA therapy by Shen et al^46^ prepared a carbomer gel incorporated with methotrexate loaded thermal responsible flexible liposomes which have shown rapid drug release under thermal conditions and increased skin permeation as compared to free methotrexate.One of the studies held by Sharma et al^47^ revealed the enhanced half-life of a drug with a significant reduction in inflammation and 1.5 folds increase in analgesic effect when studied on complete Freund’s adjuvant-induced RA rat model, with aceclofenac cocrystal complexed with lysine and incorporated into liposomes and formulated as a gel when compared to marketed gel.

 One of the important factors that affect vesicle to cross skin layers is the vesicle’s composition. Many researchers have reported various carrier systems incorporating different components in it, which plays a vital role in crossing skin barriers and enhancing permeation through stratum corneum.^48^

###  Glycerosomes

 Glycerol is widely accepted for topical application and known to access deformability index which means it modifies the fluidity of liposomal bilayer vesicle which can increase its penetration through the skin.^49^ Glycerol at a concentration of 10%-30% v/v with phospholipids and cholesterol gives rise to glycerosomes which form a flexible vesicular structure with increased penetrating power as compared to liposomes.These systems are more stable than liposomes.^50^ Salem et al^51^ conducted a study in which they have prepared celecoxib and cupferron glycerosomes for topical application, the results showed spherical morphology of drug-loaded glycerosomes with optimum particle size and high entrapment of about 89.66 ± 1.73%, 93.56 ± 2.87%, and showed significant drug release with high permeation and remarkable paw edema reduction as compared to control group. Drugs which possess limitations such as poor bioavailability on oral administration, or less lipophilic in nature, or undergo hydrolysis or efflux process can be most suitable to give via transdermal route, incorporating it in vesicular structure which promotes its penetration. One of the studies reported, by Zhang et al^52^ formulation of glycerosomes loaded with paeoniflorin (monoterpene glucoside), which possess an anti-inflammatory and immunomodulatory activity for transdermal delivery resulted in enhanced accumulation in the synovium, elevated drug concentration at knee joint for a longer period, and with good skin biocompatibility.

###  Ufasomes

 Synovial capsule barrier and skin barrier limit the efficacy of transdermal delivery. Addition of unsaturated fatty acids can overcome this barrier.^53^ As reported, unsaturated fatty acids by disrupting the tight junction or increasing the membrane fluidity can enhance skin permeation. The oleic acid and linoleic acid which are unsaturated fatty acids tend to form a vesicular structure when come in contact with the aqueous environment, these self-assembled nano-sizes are termed ufasomes.^54^ The formation of ufasomes takes place with unsaturated fats and their ionized species that are soaps and it is a pH-dependent process.^55^ The process takes place at basic pH ranging from 7-9, as vesicle formation of unionized fatty acid and fully ionized groups occurs at this pH.^56^ These nanovesicles allow lipid exchange by getting attached to the skin surface.^57^ These are capable to carry drug molecules and increasing penetration efficiency across stratum corneum.^54^ Sharma and Arora^58^ developed a gel containing oleic acid vesicle (ufasomes) loaded with methotrexate its In-vitro evaluation showed three to four folds higher permeation from that of plain gel, and 50% of the administered dose was detected in the skin from skin permeation assay.

###  Aspasomes

 Vesicular structure made up of ascorbyl palmitate in combination with cholesterol and a negatively charged lipid that is dicetylphosphate is termed as aspasomes. These carriers possess inherent antioxidant properties due to the presence of ascorbyl palmitate.^59^ Ghosh et al^60^ developed a methotrexate-loaded aspasome hydrogel which has shown in decline in rat paw diameter (21.25%), cartilage damage (84.4%), pannus formation (84.38%), inflammation (82.37%), and bone resorption (80.52%) when compared with arthritic control rat.

###  Proposomes

 Propylene glycol plays a vital role to enhance skin permeation by solubilizing stratum corneum lipids. Incorporating propylene glycol into a liposome can increase cutaneous and percutaneous drug penetration via the synergistic effect of propylene glycol and phospholipids.

 So, this type of liposome is termed proposomes. Because of its more viscous and low volatile nature, it is also found to improve the stability of liposomes.^61^ Kathuria et al^62^ developed tofacitinib (Janus kinase inhibitor) loaded proposomes for transdermal delivery and found that proposomes have shown enhanced permeation of tofacitinib by 4-11 folds and were stable for 6 months.

###  Transferosomes

 Aclass of liposomes with highly deformable, elastic, and an ultra-flexible vesicular structure composed of phospholipids such as phosphatidylcholine and edge activators like sodium cholate, deoxycholate, Span 80, Tween 80 which increases the flexibility of vesicle by destabilizing lipid bilayers.^63^ These ultra-deformable nature of vesicles increases the ease of penetration through the skin by squeezing themselves through intracellular lipids of the stratum corneum and making the drug available to a deeper layer of skin.^64^ The formation of osmotic gradient at the application site across skin, assist penetration.^65^ They possess an ability to carry hydrophilic and hydrophobic moieties and can accommodate drug molecules with a wide range of solubility.^66^ Has a potential for surface modification for active targeting and can control the drug release. All these characteristics make it an ideal carrier.^65^ In a study conducted by Yuan et al,^67^ showed the use of hygroscopic and biocompatible molecule that is Hyaluronic acid to modify the surface of transferosomes loaded with indomethacin resulted in superior drug penetration with sustaining the drug release.^67^ Preeti and Kumar^68^ investigatedsoya phosphatidylcholine and sodium deoxycholate transferosomal gel of celecoxib, a cyclooxygenase -2 inhibitor for *in vitro *and* ex vivo* drug release and *in vivo* anti-inflammatory activity showed higher permeation tendency as compared to conventional gel for the management of RA. Another study conducted by Sarwa et al^69^ found that capsaicin-loaded transferosomal gel showed 76.76% of drug release from the vesicular system in 24 hours as evident from confocal laser scanning micrographic study, with superior anti-arthritic activity as compared to marketed gel detected by *in vivo* studies.^69^

###  Ethosomes

 Ethanol affects the intercellular regions of the stratum corneum and acts as a permeation enhancer. The incorporation of ethanol in relatively high concentration with phospholipids and water forms a soft vesicular structure called an Ethosome. These carriers enhanced the delivery of drugs through the skin.^70,71^ The formation of vesicles with soft malleable structures relies on the fluidizing effect of ethanol on phospholipid bilayer. Concentrations of ethanol vary from 20-50%. As ethanol disturbs the stratum corneum bilayer by increasing its fluidity which eases the penetration of the ethosome, a fusion of those with the cell membrane in deeper skin layer triggers the drug release.^72^ Fan et al^73^ explored the efficiency of tetrandrine-loaded ethosomes and studied drug deposition and drug flux across rat skin which was 1.7 and 2.4 folds higher than that of liposomes, respectively. This indicates that ethosomes as a promising vehicle for topical drug delivery. Another study conducted by Anjum et al^74^ investigated the potential of ethosomes to deliver naproxen into deeper skin layers and, assessed the efficacy of naproxen ethosomal formulation using the carrageenan-induced rat paw edema model. Which was found to be more effective in inhibiting swelling paw edema as compared to marketed formulation. A nanoethosomal dispersion was developed for topical delivery of flufenamic acid was developed by Muslim and Maraie^75^ which resulted in formation of stable formulation, with high entrapment and sustaining the action upto 24 hours after single administration.

###  Transethosomes

 It is a combination of ethosomes and transethosomes, which shows a higher potential to permeate through skin layers. This system contains both an edge activator and ethanol which makes it easier to cross the skin barrier by squeezing through lipid layers of the stratum corneum using transdermal water gradient pressure.^56^ This system can be proven as more efficient to deliver medium to large size bioactive molecules.^76^ An edge activator (biocompatible surfactant) improves flexibility whereas ethanol deforms skin layers and adds up to vesicular fluidization.^77^ They can be loaded with both hydrophilic and lipophilic molecules.^78^ One of the studies held by Garg et al^79^ describes the preparation of piroxicam-loaded transethosomes, which were then incorporated into the gel. The optimized batch showed entrapment efficiency of 68.434% with improved stability and drug permeation. RA is also accompanied by oxidative stress because of reactive oxygen species generated by neutrophils. The study explored by Song et al^80^ to reduce this oxidative stress using transethosomes, transferosome’s surface was decorated or covered with ascorbic acid forming an antioxidant surface. These transferosomes were loaded with sinomenine hydrochloride and revealed higher drug concentration in a synovial fluid resulting in higher penetration.

###  Niosomes

 These are microscopic bilayer vesicular structures made up of a non-ionic surfactant and cholesterol arranged in such a manner that the outer and inner surface is hydrophilic area sandwiched lipophilic area between them.^81^ The non-ionic character plays vital role in drug delivery, with controlling the drug release and targeted action.^82^ Surfactant present act as a penetration enhancer by various mechanisms like altering the barrier functions of the skin, or by getting adsorbed at the interface.^83^ These vesicular carriers are biocompatible, biodegradable, relatively non-toxic, with an ability to entrap lipophilic, hydrophilic, and amphiphilic drug molecules, forming a suitable alternative to liposomal delivery.^84^ Auda et al^85^ studied the anti-inflammatory activity of the drug celecoxib from different niosomal gel formulations using the carrageenan-induced rat paw edema method, results showed that there is significant anti-inflammatory activity (75.45%) of the poloxamer niosomal gel on rat paw edema with significant drug release of 72% after 12 hours. The niosomes of liposomes have physical stability issues, drug leakage during storage, and low encapsulation efficiency, which are overcome by preparing proniosomes.^86^ Proniosomes are niosomal hybrid, which gets hydrated with water from skin layers under occlusion and get converted into niosomes, then permeates the skin barrier.^87^ These formed niosomes are very similar to conventional niosomes.^88^ Alsarra et al^89^ investigated ketorolac-loaded proniosomes and found that the niosomes produced upon hydration show 99% drug encapsulation and can be a promising vehicle for drug delivery. Ammar et al^90^ developed a tenoxicam (NSAID) proniosomal gel which is found to be non-irritant with significantly higher anti-inflammatory and analgesic effects than that of oral marketed tenoxicam tablets.

 A new form of vesicular carrier formed by addition of bile salts into a nonionic surfactant vesicles or niosomes called bilosomes.^91^ This type of vesicles is mostly preferred for oral vaccines, where the entrapped vaccine is protected from gut environment and enzymes due to presence of bile salts.^92^ Bile salts has the capability to penetrate the biological membrane, enhancing flexibility of vesicles and making it to penetrate deep layers of skin on topical application. Elkomy et al conducted a study where chitosan coated bilosomes were prepared encapsulated with berberine, an alkaloid having anti-rheumatic action. This bilosomes showed 83.8% of drug entrapment, enhanced skin permeation, with reduction in paw edema swelling.^93^

###  Lipid nanoparticles systems

 Solid lipid nanoparticles as the name indicated they are composed of single solid lipid or a mixture of solid lipid dispersed in an aqueous phase and stabilized by surfactant. The lipid phase is solid at room and body temperature.^94,95^ These are colloidal carriers with particle sizes ranging from 50 to 1000 nm, used to improve drug penetration through the skin The presence ofsolid lipid can prolong the drug release, to get sustain action. The lipids used are categorized as the “generally recognized as safe” (GRAS) category.^96^ The study conducted by Khuranaet al^97^ reported the potential of meloxicam-loaded solid lipid nanoparticles-loaded gel and evaluated for its penetration and anti-inflammatory potential and found to have a potential to transport the drug to various skin layers with marked anti-inflammatory activity and excellent skin tolerability.^97^ Theuse of pure solid lipid in solid lipid nanoparticles tends to crystallize in a perfect crystalline structure after manufacturing, resulting in the expulsion of drugs and relatively low drug loading. Nanostructure lipid carriers are the second generation of solid lipid nanoparticles, composed of a liquid lipid along with solid lipid which avoids the formation of perfect crystal lattice which reduces drug expulsion and increases drug loading.^98^ The incorporation of liquid lipid increases the possibility of efficient entrapment of a drug that is more soluble in liquid lipid.^99^ These nanoparticles form a monolayered lipid film on the skin which blocks the water evaporation due to the occlusion effect resulting in increased skin hydration which assists drug penetration.^100^ Gu Y et al^101^ developed triptolide loaded nanostructured lipid carrier for transdermal application which was evaluated for its encapsulation efficiency and drug loading, found to be 97.15% ± 9.46 and 10.35% ± 1.12, respectively with sustained release of drug and a significant reduction in knee edema in addition to marked anti-inflammatory effect by regulating the levels of TNF-α, IL-1β, and IL-6, reveled from *in vivo* studies.

###  Cubosomes

 One of the lipid nanocarrier self-assembled system with non-lamellar, mesophasic nanostructure lyotropic liquid structure called cubosomes are also found suitable for topical delivery.^102^ As the name suggests lipids bilayers are arranged in a regular cubic form separated by interconnected aqueous channels. Lipids like monoolein, monolinolein, phytantriol, etc. are used.^103^ Monoolein, a blend of oleic glycerides and other unsaturated fats is the base for cubosome development.^104^ Their unique properties like high drug entrapment, high surface area, sustained release of drug and ability to encapsulate hydrophilic, lipophilic and amphiphilic drug molecules make it a suitable carrier. The study conducted by Gorantla et al,^105^ showed developed cubosomes using glyceryl monooleate as liquid crystal structure forming material, incorporating tofacitinib is found to be suitable for transdermal application. Presence of oleic acid in glyceryl monooleate makes it more permeable through skin.^106^

 There are many more studies carried over lipid-based nanocarriers, utilizing various systems with synthetic or natural phytoconstituents to deliver the drugs through skin layers by formulating as a hydrogel, cream, emulgel, etc which are tabulated in Table 1.

**Table 1:** A study was conducted on lipid nanocarrier delivery systems for drug delivery through the skin

**Drug**	**Category**	**Carrier**	**Outcome**	**Reference**
Aceclofenac	NSAIDs	Nanoemulsion	There was a significant increase in permeability parameters like steady-state flux (Jss), permeability coefficient (Kp), and enhancement ratio (Er) with increased inhibition value of carrageenan-induced rat paw edema.	^107^
Curcumin and Emu oil	Anti-inflammatory	Nanoemulsion	A significant improvement was noted in anti-inflammatory activity which was concluded from Arthritic scoring, paw volume, biochemical, molecular, radiological, and histological examinations. The combination was more potential than only curcumin.	^108^
Celecoxib	Cyclo-oxygenase-2 inhibitor	Nanoemulsion	There was 2.97 folds increase in bioavailability with nanoemulsion gel as compared to the oral capsule. Photomicrograph of skin revealed disruption of lipid bilayers in the epidermis by nanoemulsion, resulting in enhanced permeation.	^109^
Diflunisal	NSAIDs	Nanoemulsion (nanoemulgel)	The study concluded that Nanoemulgel loaded with Diflunisal ternary complex formulated with xanthum gum significantly enhances in-vitro permeation and showed improved anti-inflammatory activity.	^110^
Celecoxib	Cyclo-oxygenase-2 Inhibitor	Liposomes	The formulated transdermal liposomes showed 81.25% of drug release in the first 24 h with a drug encapsulation of 43.24% and found to be a potential carrier for topical application.	^111^
Diclofenac	NSAIDs	Liposomes	Small unilamellar vesicular liposomes made up of soya lecithin and cholesterol incorporated into gel showed prolonged anti-inflammatory effects compared to regular diclofenac gel without liposomes.	^112^
Curcumin	Anti-inflammatory, Antioxidant.	Liposomes	Curcumin-loaded liposomal gel has the potential to reduce TNF-α expression and a direct inhibitory effect on synovium hyperplasia in RA rats.	^113^
Prednisolone	Steroidal anti-inflammatory agent	Liposomes	A proliposomal gel was prepared which has shown prolonged drug release and 60% of edema inhibition which was more than the marketed formulation.	^114^
Lornoxicam	NSAIDs	Liposomes	Lornoxicam liposomes provided a sustained release for 8 h with good skin permeation.	^115^
Triptolide	Anti-inflammatory and immune-modulator	Glycerosome	The optimized formulation was stable with an entrapment greater than 75%, with particle size 153.10 ± 2.69 nm.	^116^
Etodolac	NSAIDs	Ufasomes	The study concluded effective drug penetration with reduced toxic effects.	^117^
Dexamethasone	Anti-inflammatory	Ufasomes	Optimized formulation showed 4.7 times higher permeation than plain gel with significant anti-inflammatory activity estimated by carrageenan-induced rat paw edema model.	^118^
Curcumin	Anti-inflammatory, Antioxidant	Transferosome	The in-vivo study showed reduced pro-inflammatory cytokines through NF-κβ inhibition, with significant therapeutic efficacy.	^119^
Aceclofenac	NSAIDs	Ethosomes	Formulated ethosomes showed entrapment of 91.06 ± 0.79% and the range for cumulative drug permeation was found to be 0.26 ± 0.014 to 0.49 ± 0.032 mg/cm^2^.	^120^
Etodolac	NSAIDs	Transethosome	A transethosomes, with particle size 268.1 nm with entrapment of 83.75 ± 0.61% loaded into gel showed in vitro drug release of 97.35 ± 0.43% when studied for 12 h.	^121^
Thiocolchicoside	Anti-inflammatory and analgesic	Niosomes	Topical thiocolchicoside niosomal gel showed controlled drug release with increased topical retention time which has reduced dosing frequency and side effects.	^122^
Piroxicam	NSAIDs	Solid lipid nanoparticles	The formulated SLN gel exhibited increased skin permeation as compared to the marketed gel.	^123^
Lornoxicam	NSAID	Nanostructured lipid carrier (NLC)	NLC loaded gel showed 48.77% swelling inhibition after 6 hr. when Carrageenan induced paw edema anti-inflammation activity was carried out.	^124^
Ibuprofen	NSAID	NLC	Mean particle size was 160 nm with entrapment of about 98.15% and showed greater potential to enhance drug penetration.	^125^

###  In vivo animal models for rheumatoid arthritis

 To recognize drug targets for RA and test potential therapeutics animal models of arthritis are widely used.^126^ They represent an important tool for exploring and evaluating new treatment options and contribute to descriptive and in-vitro studies. Collagen-induced arthritis is the most widely used model as it required both T-cells and B-cells to autologous collagen II for disease manifestation and show immunological and pathological features like human RA.^127^ The other animal models used are summarized in Table 2.

**Table 2:** Commonly used Animal Models for Rheumatoid Arthritis.

**Animal Model**	**Species**	**Antigen **	**Adjuvant**	**Involvement of Immune system**	**Reference**
Collagen-induced arthritis	Rat	Type II collagen	Incomplete Freund's adjuvant or complete Freund's adjuvant	T-cells, B-cells, complement, Major histocompatibility complex (MHC), Monocytes	^126,127^
	Mice	Type II collagen	Complete Freund's adjuvant	T-cells, B-cells, complement, MHC, monocytes	
Adjuvant-induced arthritis	Rats	-	Components of mycobacteria, muramyl dipeptide, or incomplete Freund's adjuvant	CD4 + T cells, B-cells, macrophages, granulocytes, dendritic cells.	^126,127,128^
Antigen-induced arthritis	Mice/Rat/ Rabbit	Methylated bovine serum albumin	-	T-cells, B-cells, complement, monocytes, dendritic cells	^128,127^
Streptococcal cell wall induced arthritis	Rat	-	Bacterial cell wall fragments	T-cells, B-cells, MHC, monocytes.	^129,128^
Proteoglycan-induced arthritis	Mice	A human fetal cartilage proteoglycan	-	T-cells, B-cells, Complement, MHC	^129,126^
Pristane induced arthritis	Mice/Rat	-	2,6,10,14-Tetramethyl pentadecane (pristane)	T-cells, B-cells, complement, MHC	^128,129^
Spontaneous models (Human TNF-alpha transgenic mice model)	Mice	Overexpression of Tumor necrosis factor-alpha	-	Monocytes, granulocytes.	^130,126^

## Patents granted for rheumatoid arthritis

 Several patents are been filed worldwide on treatment or diagnostic approaches for RA. Table 3 includes patents filed by various countries like the United States, Europe, Australia, China, etc.

**Table 3:** Patents on rheumatoid arthritis

**Publication Number**	**Publication date**	**Assignee/Inventor**	**Patent Title**	**Reference**
**Patents On Nanosystems Approach in RA**				
US20090232731A1	2009-09-17	Medigene AG	“Cationic Liposomal Preparations for the Treatment of Rheumatoid Arthritis”	^130^
US10709664B2	2020-07-14	Yale University	“Nanolipogel comprising a polymeric matrix and a lipid shell”	^131^
US8715736B2	2014-05-06	Florida Agricultural and Mechanical University FAMU	“Nanoparticle formulations for skin delivery”	^132^
AU2021106679A4	2021-12-02	K. K. Sahu Shikha Shrivastava Deependra Singh Manju Rawat Singh	Enzyme-loaded navigated nanomatrix systems to the inflamed synovial locus for the treatment of rheumatoid arthritis.	^133^
**Patents related to other treatment approaches for RA**				
US11195595B2	2021-12-07	Scipher Medicine Corp.	“Method of treating a subject suffering from rheumatoid arthritis with anti-TNF therapy based on a trained machine learning classifier.”	^134^
US20170157249A1	2017-06-08	AbbVie Biotechnology Ltd	“Uses and compositions for treatment of rheumatoid arthritis”	^135^
US20210230719A1	2021-07-29	Sanofi Biotechnology SAS Regeneron Pharmaceuticals Inc	“Compositions for the treatment of rheumatoid arthritis and methods of using same”	^136^
US10913792B2	2021-02-09	Morphosys AG	“Treatment for rheumatoid arthritis.”	^137^
EP3102575B1	2021-01-13	Galapagos NV	“Novel salts and pharmaceutical compositions thereof for the treatment of inflammatory disorders.”	^138^
EP2680884B1	2018-01-17	F Hoffmann La Roche AG	“Biological markers and methods for predicting response to b-cell antagonists.”	^139^
CN102597268B	2017-09-22	F Hoffmann La Roche AG	“For a treat, diagnose and monitoring rheumatoid arthritis method”	^140^
CA2760460C	2019-04-02	Zynerba Pharmaceuticals Inc	Transdermal formulations of cannabidiol comprising a penetration enhancer and methods of using the same.	^141^
AU2018260845B2	2020-09-03	Takeda Pharmaceutical Co Ltd	“Evaluation and treatment of bradykinin-mediated disorders”	^142^

## Conclusion

 Although there are several drugs available for use in RA treatment, however, none of the present pharmacotherapies have proven to be efficient in treating RA; they have several flaws that limit their efficiency. Drug delivery through the skin can be a promising strategy for the management of RA which has offered enticing possibilities for addressing the low bioavailability of certain oral medications, several side effects, and injection pain and discomfort. This review aimed to give an outline of the use of various lipid nanocarrier systems, including liposomes, nanoemulsion, niosomes, nanostructured solid lipid carriers, and some modified carries with ethanol, glycerol, propylene glycol, etc. which adds specific characteristics and flexibility to potentially cross skin barrier improving drug penetration. Liposomes shows high drug loading for water-insoluble drugs. On the other side noisome are resistance to oxidation forming a stable carrier. Whereas ultradeformable carriers such as transferosomes, ethosomes, transethosomes can easily penetrate through stratum corneum, addition of penetration enhancers like oleic acid, propylene glycol, glycerol can also enhance the penetration ability through skin. Formulation of solid lipid nanocarriers enhance stability and improve drug entrapment than other carrier systems. Various studies conducted, showed the biocompatible nature of lipid nanocarriers with minimal toxicity and high drug entrapment provides high therapeutic efficacy. After considering the issues raised concerning the adverse effect of drugs given through conventional routes and crossing skin barriers, one can conclude that the use of these lipid nanocarriers for drug administration through the skin is a valuable and promising approach. The review also highlights various studies conducted, giving a brief overview regarding the use of in-vivo arthritis models.

## Competing Interests

 The authors declare that there are no conflicts of interest. All co-authors have seen and agree with the contents of the manuscript and there is no financial interest to report.

## Ethical Approval

 Not applicable.
